# Developing a scalable high-value solution for cancer care and control in India

**DOI:** 10.7189/jogh.16.04064

**Published:** 2026-02-20

**Authors:** Amrit Virk, Che L Reddy, Dinesh Pendharkar, Rifat Atun

**Affiliations:** 1 Department of Social Policy, University of Edinburgh, Edinburgh, UK; 2 Health Systems Innovation Lab, Department of Global Health and Population, Harvard T.H. Chan School of Public Health, Harvard University, Boston, Massachusetts, USA; 3 Medical-Hemato-oncology, BMT, Cell & Gene Therapy, Sarvodaya Cancer Institute, Faridabad, India; 4 Department of Health Policy and Management, Harvard T.H. Chan School of Public Health, Harvard University, Boston, Massachusetts, USA; 5 Department of Global Health and Social Medicine, Harvard Medical School, Harvard University, Boston, Massacsusetts, USA; **Background** India faces major health system gaps in screening, diagnosis, and treatment for cancer control. Context-sensitive, frugal models of service delivery – often characterised as jugaad in India for their emphasis on pragmatic, cost-efficient problem-solving and solution design – have emerged to address barriers in resource-constrained health systems. We analysed an innovation designed to expand screening, accelerate referral pathways, and strengthen access to specialist cancer care in India.; **Methods** We conducted an exploratory qualitative study using documentary analysis and structured interviews with the innovation founder. Data were examined using an established complex innovation-diffusion analytical framework to characterise the model, assess factors influencing adoption across states, and identify enablers and constraints shaping scale-up. The primary endpoint was to examine the design features, approach to introduction in the health system, and system interactions through which the model improved the effectiveness, efficiency, equity, and responsiveness of health services for cancer control.; **Results** The innovation emerged through pragmatic, frugal problem-solving and comprised three integrated components: (i) a low-friction district cancer unit established by repurposing existing space, equipment, and staff, (ii) a practice-centred oncology upskilling model that enabled medical officers and nurses to deliver oncology services with minimal changes to existing roles, and (iii) a low-touch digital clinician decision support channel that provided real-time specialist input through a simple smartphone messaging platform. Gradual adoption occurred in six states, driven by political champions, alignment with state health system priorities, and the ‘low-touch, low-friction’ design of the innovation. However, limitations to scale-up include reliance on individual leadership, variability in training, fragmented digital systems, suboptimal engagement with policymakers, and the absence of routine outcome measurement systems.; **Conclusions** This exploratory study highlights how frugal, adaptive models can enhance the effectiveness, efficiency, equity, and responsiveness of health services for cancer control in constrained health system contexts such as India. However, it also shows that long-term sustainability and scale-up will require formalised training, dedicated financing, digital integration, an inclusive, independent, and diversified governance structure beyond the individual innovator, federal-level political support, and independent outcome measurement. Future research should include comparative multi-state analyses and routine incorporation of standardised indicators to quantify the effect on cancer control and scalability.

Globally, health systems are underperforming in their cancer control response, and India is no exception [1]. India is experiencing accelerating cancer incidence and age-standardised mortality [2] with major disparities in relation to household income, geography, and gender [3]. Major gaps in cancer control and uneven distribution of facilities exacerbate challenges to care and outcomes produced. Large swathes of India’s population (52% of rural households) are dependent on public sector health services [4], overrun and underequipped to cope with the scale of demand. Where infrastructure exists, social and cultural factors, including fear, stigma, gender roles, and low awareness may undermine cancer control efforts.

A major opportunity exists to develop innovations that enhance country's cancer control response. We take an expansive view of ‘cancer control’ to include all ‘health system actions, including public health and individual health service interventions across the care continuum, aimed at reducing the incidence, morbidity, suffering and mortality of cancer and improving the survival and well-being of individuals with cancer’ [5]. We define a high-value health service in relation to cancer as one that is effective, efficient, equitable, and responsive [6]. While novel therapeutic technologies can enhance effectiveness of cancer control and are often costly, innovations in care delivery are less well documented but have the potential to harness existing technologies to enhance efficiency, equity, and responsiveness. Knowledge of frugal innovations that are cost-efficient, responsive to patient needs, and capable of adoption at scale in health systems could provide lessons to expand cancer control in similar settings. This study documents one such cost-efficient cancer control innovation in India, utilising an innovation and diffusion framework to explore the potential of frugal, adaptive models for improving access to health services for cancer control in constrained health system contexts. The model reflects features of frugal, resource-constrained innovation commonly described in India as jugaad, characterised by iterative adaptation, pragmatic problem-solving, and efficient use of existing system assets.

The paper is organised in four sections. In the next section, we describe the theoretical framework and methodology. Section 2 offers context, outlining the cancer burden and current response in India. Section 3 examines the key features, barriers, and facilitators for the innovation, with Section 4 distilling key lessons for policy and practice, with reflection on the directions for future work.

## METHODS

This case study of a local cancer control innovation in India was a component of a larger project documenting India’s cancer care programme. An exploratory, qualitative approach [7] was employed utilising documentary analysis and semi-structured interviews with the innovation founder. As a novel, under-researched case with limited information related to it, a pragmatic approach was necessary to source any relevant information that was publicly available or obtainable from the key informant [8]. Purposive and convenience techniques were used to identify key documents that reported on and directly related to the innovation complemented with in-depth interviews with the innovation founder. Guided by our analytical framework (**Box 1**), a topic guide was prepared to execute three sets of exploratory expert interviews with the innovation founder (DP), a lead oncologist who pioneered the care model. These ranged from 30 minutes to over an hour conducted between February and March 2023 via Zoom by the lead researcher (AV). Our aim to distil and document the key features of the programme resulted in limiting interviews to a single informant, although further analysis and evaluation would call for a larger sample of interviewees. Documents included available published information as well as grey literature obtained online or sourced from the key informant such as state government operational guidelines, meeting notes, video presentations, commentaries, as well as programme publicity and promotional materials.

Box 1 **Definitions and analytical framing**

Innovations in health systems are progressive ideas, tools, and techniques to confront new and persisting problems faced by health systems. They are engineered to respond to the inherent dynamism and adaptability of health systems as they seek to bring about positive changes to the system's performance to improve patients' lives. Policies, medicines, diagnostic technologies, institutional arrangements, processes, and practices perceived as novel or new by the adopting entities all fall under the innovation umbrella. Additional to an innovation's design and structure, its dynamic interactions with the health system and wider health ecosystem are often crucial for its expansion and endurance.

An innovation and diffusion lens [6] informed the analysis. The innovation and diffusion framework enabled appraisal of the complex interplay between the care model, key actors, the health system, and setting, to situate the technical design of the model within a real-world context. This allowed for a more nuanced appraisal of the innovation's progress and prospects rather than a simple description detached from reality.

### Study limitations

Using grey literature and non-standardised reports from the field meant information gaps and challenges with drawing definitive conclusions on impact and effectiveness. Independent verification through administrative data or third-party audits was not possible and should be a priority for future research. Similarly, leaning on the account of the innovator is a limitation, introducing risks such as confirmation bias. Limited contemporaneous documentation for earlier phases of the programme and relying on the founder’s account also introduces the risk of recall bias. However, we sought to minimise these risks through:

(i) cross checking across data sources and publicly available information to the extent possible

(ii) independent coding and analysis of information (AV), validation of interpretations by a second researcher (CR), followed by further ratification by a third researcher (RA).

While acknowledging these limitations to imputing the innovation’s impact, this study makes a vital contribution in chronicling a service delivery innovation in a low resource setting, its survival and progression testifying to its potential.

### Data analysis

Data gathering was halted once sufficient data had been obtained to document the context for the emergence, key features, and processes related to the innovation [9]. Online transcripts were generated and checked by the lead author for accuracy. Data analysis was iterative, involving re-reading texts and listening to interview recordings to code and derive relevant themes, using a ‘best fit’ framework synthesis approach [10]. The best fit framework involves the development of an ‘a priori framework’ that offers a tool for rapid analysis to describe or explain a phenomenon and hence was suited to our study aim focused on describing the emergence of an innovation for cancer control within a mixed health system with public and private provisioning. An in-house case study template used for pedagogical purposes was used by the lead author to develop an initial analytical frame, which was revised and elaborated in discussion with the co-authors (CLR, RA) to develop the *a priori* framework broadly guided by the innovation and diffusion approach underpinning our analysis, *e.g.* the innovation, innovation components, the adoption system, institutional environment, health system characteristics, broad context, introducing the innovation, scaling the innovations, sustainability challenge, where next (**Box 1**).

## RESULTS

### The problem: rising cancer burden in India and suboptimal system performance

One in nine Indians are anticipated to develop cancer in their lifetime, with ratios as high as 1 in 68 males (lung cancer) and 1 in 29 females (breast cancer) [11]. Cancer incidence and mortality rates have risen sharply, increasing 2-fold from 1990 to 2016, and the age-standardised mortality rate was 64.4 per 100 000 in 2022 [2]. Breast, cervix uteri, and ovarian cancers are more frequently diagnosed among women, whereas lip, oral cavity, lung, and oesophagus cancers are more commonly seen among new cases among men in India [2].

Cancer incidence, mortality, and five-year survival rates rely on health system management and performance – reflecting the degree to which the health system can deliver effective, efficient, responsive, and equitable cancer control services including screening, diagnosis, treatment, and palliative services. As with health care generally, cancer control in India reflects enduring problems of poor uptake, access, and availability of services contributing to high levels of under-diagnoses and under-treatment [12]. Care seekers lack a clear referral pathway and are likely to bypass primary care services in favour of larger, district hospitals or alternatively private sector providers. Despite notable policy efforts directed towards expanding cancer prevention and treatment, cancer control services are mainly located within hospitals and urban areas rather than at entry-level point of care and rural areas [13]. Similarly, 60% of specialist centres are in the country’s Southern and Western regions, at a great distance from the Northeastern states which report some of the highest cancer incidence rates [3]. Moreover, there are severe resource shortages in the production and distribution of human resources, the number of health facilities, infrastructure, supplies and pharmaceutical products for cancer: a recent government report testifies to a severe shortfall in radiotherapy equipment, 0.54 per million people in India *vs*. the WHO norm of one per million population [14].

On financing, cancer patients in India shoulder a massive financial burden, with roughly three-quarters of cancer care paid for out of pocket by patients [15]. Inpatient care constitutes over a third (37%) and outpatient care 49% of a household's monthly consumption expenditure in India [16]. Out of pocket spending poses a heavier burden on poorer population groups, older populations over 60 years, and those in rural areas, signalling further inequities in current financing mechanisms for cancer in India. Except for two states, private cancer care is generally costlier than at public cancer centres nation-wide. Whereas average individual payments amount to Indian Rupee (INR) 64 977 (approximately 818 USD) at public facilities, private facilities could cost over twice as much at INR 133 464 (approximately 1679 USD) [17]. Despite costlier care, cancer patients seek out private facilities for both inpatient and ambulatory services [16].

### The innovation: conception, design, and introduction

The control model was introduced in 2015 in a single state integrated into its public cancer programme, gradually extending further. It was designed to strengthen cancer control and delivery in government-run district hospitals and streamline care pathways by creating niche cancer care units within district hospitals, led by general doctors who were upskilled to be point personnel for care seekers. It thereby reoriented the state government’s role towards stewarding a comprehensive cancer control programme, providing a customised space and personnel for potential patients. Patients coming to district hospitals fell into three broad categories:

(i) suspected cancer patients,

(ii) confirmed cancer patients diagnosed at private facilities who could not afford the cost of private treatment, and

(iii) more advanced cancer patients who had received initial treatment at other facilities but could no longer pay for further treatment.

Effectively, through the government’s cancer control programme, patients had a focal location and point person to reach. The general duty medical officers who functioned as nodal cancer officers at district hospitals offered care continuity as well as help to patients to navigate the next stage of care (either in government hospitals or at private facilities of the patient’s choosing).

### Major components of the innovation

#### Component 1: low-friction district cancer unit

This component created a cost-efficient district cancer care unit by repurposing existing space, equipment, and staff, enabling delivery of essential cancer services through a ‘low friction’ model that integrated seamlessly into routine hospital workflows.

The delivery mechanism was embedded in existing clinical workflows and consisted of:

(i) A new nodal cancer care unit in each district hospital creating an earmarked space to provide necessary cancer services.

(ii) Resourcing each cancer unit with necessary supplies to provide an essential package of care including consultations with doctors, intravenous fluids, painkillers, and anti-cancer drugs. Basic investigations like x-rays and blood tests were usually available at district hospitals, albeit with some variation in facility capacity. More advanced procedures were made available through state governments’ arrangements with external laboratories, alongside ongoing government efforts to better equip district hospitals.

(iii) An in-house doctor and nurse team were trained in practical skills to function as ‘patient navigators’ within the unit and to function as the interface between patients and specialist oncologist services.

A general duty medical officer (GDMO), one of four sub-cadres of publicly employed doctors in government-run hospitals in India [18], typically doctors with basic medical qualifications in general practice, as well as two nursing staff from each district hospital were identified to undergo a rapid, one-on-one oncology learning exercise. The doctor-nurse teams received basic training on a range of critical aspects of cancer control including diagnosis, chemotherapy administration, management of side effects, consultation, and counselling, effectively reprofiling existing roles for providers to respond more effectively to patients. The upskilling of existing personnel and the development of a cadre of versatile, general doctors to manage shortages of specialist doctors in government hospitals was a unique selling point to convince state bureaucrats of the model’s merits.

#### Component 2: practice-centred oncology upskilling model

This component provided a one-month practical training module that upskilled existing general-duty medical officers and nurses to perform basic oncology services, adding new skills and knowledge without major changes to existing service arrangements.

Unlike traditional oncology training modules that focus on theoretical knowledge of cancer and its cellular biology, this programme’s training module was uniquely practice oriented. State-run cancer care programmes corralled medical officers on general duty (GDMO), the fulcrum of district hospital’s day-to-day administration, to provide a range of screening, chemotherapy, and palliative care services. The month-long experiential training module focused on getting trainee doctors to counsel a cancer patient and learning to read and synthesise an oncology report for a specialist oncologist to advise on the next course of action. Initial training was structured as a dialogue between trainee General Duty Medical Officers (GDMOs) and the specialist oncologist/trainer (innovation founder), followed by practical discussions on actual patient reports, learning to identify patient pathways, note taking, and asking clarifying questions. History taking and timelines for when cancer was diagnosed, its location and spread, and progression over time were considered sufficient to craft a ‘10-line story’ for a specialist oncologist to determine the form of cancer. A trimmed-down, one-month module also made it easier to persuade district hospitals to release key hospital personnel with busy routines for a relatively short time while giving them an added skill in dealing with patients they routinely attended to.

#### Component 3: digital clinical support channel

This introduced a ‘low touch’ smartphone messaging platform to offer real-time specialist support by leveraging existing communication tools already used by providers in the health system.

The programme made innovative use of mobile technology and social media (WhatsApp application) to operate a professional networking and peer support model whereby general doctors at district hospitals could connect with a cancer specialist. Trainees have the option of speaking with or messaging the innovation founder/oncologist for clarification, to arrange a virtual consultation, and to access a virtual Tumour Board for further analysis.

### Inception and rapid scale-up: the role of policy champions and networks

The prototype for this cancer control model with three integrated components that was later incorporated into states’ cancer programmes was initiated on a smaller scale in 2003–04 at the National Railways department-run hospital in the country’s capital, Delhi, which to the present day provides approximately 100 chemotherapies daily without the presence of an oncologist. While then working as a part-time consultant at the Railways hospital, the innovation founder oversaw the establishment of a day care centre to offer in-house care to cancer patients. Later, in 2014, at the behest of the head of the district hospital in Ujjain (Madhya Pradesh state), a similar cancer control delivery model was trialled even though the state government could not be persuaded to extend it more widely to other district hospitals. Shortly after, in 2015, the then state Chief Minister’s decision to prioritise cancer control and provide free anti-cancer drugs at public hospitals provided a window of opportunity for the model’s expansion. Top level attention to cancer control led to swift policy action with a senior civil servant, the Health Secretary, being charged with identifying cost-efficient options to scale-up cancer control services. Of all those approached in specialist cancer units across India, the relatively less time and resource-intensive approach being implemented in the Ujjain district hospital proved a particular draw for government officials. Within a year of its initial launch in 2014, the care model was thereby expanded and implemented across all 52 district hospitals in Madhya Pradesh that began to offer day-care chemotherapy services for eligible users.

In contrast, however, state administrators in states like Odisha, Himachal Pradesh, and Rajasthan were more cautious. While their interest was piqued at a 2017 central health ministry conference where the Madhya Pradesh government presented their cancer programme as a local initiative, civil servants and senior public sector doctors from these states remained sceptical. Subsequent site visits dissipated some of their concerns as did personal appeals from senior personnel and central health ministry officials. Hence, networking events, site visits, as well as personal appeals were three key tactics that generated understanding and receptivity towards the service delivery model already operational in Madhya Pradesh and its subsequent extension to district hospitals in a further five states (for a total of six states) covering a population of approximately 380 million.

### Enablers for scaling up

Key policy champions invested with formal authority were crucial to both the initiation and extension of this care delivery model. Policy networks of government administrators along with a few select district hospital administrators were core advocates who initiated the programme in specific districts and eventually expanded it to other districts. These champions exhibit the importance of a ‘first mover group’ that is willing to take risks and is crucial for the adoption of an innovation [6]. We also find that high level political support in Odisha, Himachal Pradesh, and Rajasthan for instance, forestalled potential resistance from within each state’s medical community, particularly from private providers who anticipated potential revenue losses from a change in status quo.

Moreover, existing public sector infrastructure and personnel propelled the care model’s introduction within states. This concurrence between pre-existing district hospital resources and the innovation’s two core components *i.e.* its simple design features and training protocol, all further ensured local receptivity to its adoption [6]. The feasibility and success of the care model in early adopter states, gave further momentum to discussions with more sceptical government administrators. National health secretaries who are senior civil servants within the Indian administration were approached by the innovator to persuade hesitant counterparts in key states. Another strategy deployed was to garner support from both the top-most official in the state’s administration as well as their deputy to ensure consistent formal backing for the care model in case of officials being deputed to other locations. The care model’s perceived success within policy circles has hence helped it endure despite state governments changing hands (**Box 2**).

Box 2 **Worked example to appraise the politics of innovation**

A condensed framework to appraise the political dimensions that underlie the introduction of a health services innovation.

***Study the policy environment:*** The 2010 National Programme for Prevention and Control of Cancer, Diabetes, Cardiovascular Diseases, and Stroke (NPCDCS) signalled greater policy prioritisation on non-communicable diseases. In 2016, the national government announced nationwide population-based screening programmes for cervical, breast, and oral cancers as part of the NPCDCS and another national initiative, Ayushman Bharat Comprehensive Primary Health Care Programme [19], although these have faced continuing challenges with low uptake and uneven geographic participation [20]. High level policy attention has been reflected in state governments' interest in expansion of cancer control within their territories, with State Illness Assistance Funds in operation in certain states.

***Consider the key players:*** In this case, two key institutional players comprise the adoption system for the innovation: individual state governments/state health departments under whose ambit the cancer programme runs and the district hospitals comprising the operational units for the innovation. Senior government bureaucrats (joint secretary health), hospital administration staff, and trainee medical professionals, typically a general medicine doctor and two nurses make up the key functionaries within the adoption system on the supply side.

***Devise suitable line of action:*** This would require considering sources of authority and power relations that govern the enactment of health policies generally. Influential actors in positions of authority (state civil servants, district hospital administrators) and those with medical expertise imbued personal appeals to state governments with a degree of authority and credibility. Regular site visits for consistent messaging and engaging with pre-existing policy networks of government actors created a powerful synergy for the care model's implementation.

## DISCUSSION

Three major factors explain the rapid uptake of this high-value solution to enhance equity, efficiency, and responsiveness in relation to cancer care and control for nearly 380 million people across adopting states. These include:

(i) the nature of the solution that involved a scalable and low-friction delivery model for adopters;

(ii) harnessing existing public sector infrastructure, and

(iii) canvassing political support to scale innovation by aligning the policy environment, key players, and devising a suitable line of action.

We consider each of these below while also drawing attention to deficiencies in the model’s current formulation that hindered nationwide scale-up.

### Low friction and scalable delivery model

Besides creating a physical space for cancer care and control, the model involved a cancer training plan that was atypical in its content and delivery. Unlike routine and highly technical training modules, the approach here was pragmatic and practice oriented. The model’s distinctiveness hence lies in its realistic, hands-on approach to training general medical officers in relevant district hospitals in recognition of the work and time pressures on specialist oncologists, trainee doctors, and nurses, who often had multiple responsibilities to undertake as part of their jobs. In terms of content, the training focused on getting trainee doctors to spot signs of cancer, establish its progression, and advise care seekers appropriately. The thinking behind a relatively pared down, shorter month-long training also stemmed from a realistic assessment of trainee doctors’ qualifications as general doctors, current demands on their time in managing multiple roles as hospital functionaries, and a relatively moderate proportion of care seekers needing cancer services.

### Harnessing existing public sector infrastructure

The model’s institutional logic is founded on recognising the (geographical and financial) reach, accessibility, and centrality of the public sector to any programme seeking to extend medical access and improve service delivery. State cancer control programmes were delivered through an already existing network of publicly run district hospitals that provide secondary and routine specialist services for a defined area and population and are mainly accessed by less privileged groups. The care model capitalised on existing institutional resources and the pre-established setup in district hospitals that included a state government-appointed district nodal cancer doctor.

### Canvassing political support

The employment of a crucial political approach was evident in the innovator seeking to create a receptive environment for the introduction and scale-up of the care delivery model. We found that dissemination meetings and civil servant networks were influential factors in garnering and sustaining policy interest. Personal and repeated advocacy efforts with decision-makers played a pivotal role in overcoming hesitation and convincing them about the viability and utility of the model. An ability to seize opportunities, recognising and acting upon ministerial interest in cancer control, as well as regular pitches to relevant government stakeholders, were vital to garner political support for the innovation. Along with high-level backing, continuing stewardship through a senior cadre of civil servants proved vital for the programme's continuation. While high-level government support mandated district hospitals to generally comply, individual doctors’ buy-in was critical for the programme’s stability. Appealing to doctors’ intrinsic motivations [21], principally a desire for self-determination and autonomy, the model offered general doctors in district hospitals an enhanced professional profile.

### Considerations for future expansion

The care model has enabled system strengthening by spearheading new operational guidelines for day-care chemotherapy centres and palliative care services, bringing about the inclusion of chemotherapy medicines within pre-existing essential medicine lists. More advanced diagnostic scans and specialised biomarker bloods testing were initially outsourced to external laboratories and private facilities. Over time, however, an increasing number of district hospitals (*e.g*. in Madhya Pradesh, Himachal Pradesh) have begun to offer CT scans and other advanced procedures in-house.

The increase in patient numbers is a testament to the innovation’s role in augmenting patient demand and facilitating earlier clinical intervention for cancer care (**Table 1**). This can be important for addressing historic problems with poor care-seeking practices, which lead to large numbers in India going un-diagnosed or presenting at more advanced stages of cancer [20]. Government directives in certain states have advised district hospitals to have explicit signage. Albeit ad hoc, a few instances of awareness raising by community-level auxiliary workers (ASHA) as part of routine activities as well as ongoing monthly camps at district hospitals inviting patients to pre-register for cancer care services, voluntarily providing medical staff with a record of their medical and treatment history are emerging.

**Table 1:** Number of cancer patient consultations in selected Indian states, 2019–2024

States, n	Year				
	2019–2020	2020–21	2021–22	2022–23	2023–24 (Apr–Nov 2023)
Chhattisgarh (consultation on chemotherapy)	n.d.	305	932	1178	2418
Odisha	4983	5982	7769	8924	8280
Madhya Pradesh (new patients seen)	5998	6001	5732	6018	5945
Himachal Pradesh	n.d.	n.d.	n.d.	n.d.	1198 (cumulative 14 574)

n.d. – no data

That said, it is also important to recognise the model’s limitations in its current version to suitably assess areas for improvements and its potential for scale up. Systemic challenges such as financial and resource constraints call into question the sustainability of service enhancements within district hospitals that may require localised partnership and resource mobilisation enabled by a supportive administration. Against existing capacity constraints, the implications of mobilising care seeking and then managing increased patient loads needs careful thought as this will likely place pressures on already strained staff and resources. The contained geographical reach of the programme so far has allowed the innovator himself to deliver the training module, much of it rudimentary with limited protocols, standardisation, and guidance for replication on a larger scale. Relatedly, use of WhatsApp as a clinical communication tool raises concerns about protecting patient privacy and data security that demands formal systems for regulatory compliance. A standardised curriculum, competency assessment, and follow up mechanisms will need to be developed to enable both replication on a larger scale including delivery by other trainers. It is critical that formal governance mechanisms are adopted such that through a representative, formal board and operational management team such as the programme is not reliant on a single individual. This will be crucial to ensuring independent oversight, monitoring and evaluation, and quality standards to inform further scale-up.

## CONCLUSIONS

In documenting the innovation, this study provides a baseline for future work while highlighting the need for outcome measures to appraise the model’s efficacy in meeting central health system goals and expanding access. This includes tracking long term follow up data and formal cost-effectiveness analysis. While a comparative analysis of implementation trajectories and relative success across states was outside the ambit of this study, this is a ripe area for further research into differential rates of adoption, challenges, identifiable patterns for more generalisable conclusions for similar settings as well as other non-communicable diseases (NCDs). Additionally, the effects on care seekers in terms of access to services and the role of community support in sustaining innovations requires further research.

Seen through a health systems lens, the model’s achievements lie in being responsive to local needs and progressing equity goals in enabling access to cancer care for patients at public facilities that are relatively closer and free at the point of care. While not formally appraised, the model also presents an opportunity to realise cost and efficiency goals by identifying and diagnosing patients earlier in the progression of the disease. The care model’s current configuration is generally compatible with existing public delivery structures for health, available infrastructure, and personnel, and to that extent, offers a foundation for a self-sustaining innovation ecosystem.
